# The use of instrumented gait analysis for individually tailored interdisciplinary interventions in children with cerebral palsy: a randomised controlled trial protocol

**DOI:** 10.1186/s12887-015-0520-7

**Published:** 2015-12-07

**Authors:** Helle Mätzke Rasmussen, Niels Wisbech Pedersen, Søren Overgaard, Lars Kjaersgaard Hansen, Ulrike Dunkhase-Heinl, Yanko Petkov, Vilhelm Engell, Richard Baker, Anders Holsgaard-Larsen

**Affiliations:** 1 Department of Orthopaedic Surgery and Traumatology, Odense University Hospital, Odense, Denmark; 2 Institute of Clinical Research, University of Southern Denmark, Odense, Denmark; 3 H.C. Andersen Children’s Hospital, Odense University Hospital, ᅟOdense, Denmark; 4 Department of Paediatrics, Lillebaelt Hospital, Kolding, Denmark; 5 Department of Paediatrics, Hospital of Southern Jutland, ᅟAabenraa, Denmark; 6 Department of Paediatrics, Hospital of Western Jutland, ᅟEsbjerg, Denmark; 7 University of Salford, Manchester, United Kingdom

**Keywords:** Gait analysis, Cerebral Palsy, Gait Deviation Index, Study protocol

## Abstract

**Background:**

Children with cerebral palsy (CP) often have an altered gait. Orthopaedic surgery, spasticity management, physical therapy and orthotics are used to improve the gait. Interventions are individually tailored and are planned on the basis of clinical examinations and standardised measurements to assess walking (‘care as usual’). However, these measurements do not describe features in the gait that reflect underlying neuro-musculoskeletal impairments. This can be done with 3-dimensional instrumented gait analysis (IGA). The aim of this study is to test the hypothesis that improvements in gait following individually tailored interventions when IGA is used are superior to those following ‘care as usual’.

**Methods/Design:**

A prospective, single blind, randomised, parallel group study will be conducted. Children aged 5 to 8 years with spastic CP, classified at Gross Motor Function Classification System levels I or II, will be included. The interventions under investigation are: 1) individually tailored interdisciplinary interventions based on the use of IGA, and 2) ‘care as usual’. The *primary* outcome is gait measured by the Gait Deviation Index. *Secondary* outcome measures are: walking performance (1-min walk test) and patient-reported outcomes of functional mobility (Pediatric Evaluation of Disability Inventory), health-related quality of life (The Pediatric Quality of Life Inventory Cerebral Palsy Module) and overall health, pain and participation (The Pediatric Outcome Data Collection Instrument). The primary endpoint for assessing the outcome of the two interventions will be 52 weeks after start of intervention. A follow up will also be performed at 26 weeks; however, exclusively for the patient-reported outcomes.

**Discussion:**

To our knowledge, this is the first randomised controlled trial comparing the effects of an individually tailored interdisciplinary intervention based on the use of IGA versus ‘care as usual’ in children with CP. Consequently, the study will provide novel evidence for the use of IGA.

**Trial registration:**

Trial registration: ClinicalTrials.gov NCT02160457. Registered June 2, 2014.

## Background

Cerebral palsy (CP) is a diagnosis that includes a range of conditions caused by a non-progressive brain injury occurring in the developing foetal or infant brain. Although the brain injury is non-progressive, the neuro-musculoskeletal and movement-related functions may deteriorate and cause activity limitation [1]. Most children with CP exhibit an altered gait such as stiff knee gait, crouch gait, excessive hip flexion, intoeing or equinus [2]. Thirty-eight to sixty-five per cent of all children with CP walk independently and are consequently classified on the Gross Motor Function Classification System (GMFCS) at level I or II [3, 4].

The interdisciplinary interventions addressing impairments that affect the patients’ gait can be described in four categories: orthopaedic surgery, spasticity management, physical therapy and orthotics [5, 6]. Guided by the problems faced by each child with CP, interventions should be individually planned to help the child and family to achieve their goals [6].

In Denmark, a patient-centred and evidence-based approach is pursued. An adapted version of the Swedish Cerebral Palsy follow-Up Program is used, where the healthcare professionals use standardised examinations of the child throughout childhood [7]. A local team, which usually consists of a paediatrician, a paediatric orthopaedic surgeon and a physiotherapist, is responsible for the follow up and individually tailored interdisciplinary interventions for each child with CP. The local team meets with the child and family once or twice a year to examine the child’s development and to plan and coordinate common goals and interventions for the child. As part of the Cerebral Palsy follow-Up Program, the overall gross motor function and walking performance are evaluated by standardised measures such as the GMFCS, the Functional Mobility Scale and sometimes the Gross Motor Function Measure (GMFM) [8–10]. However, objective features in the gait that reflect underlying neuro-musculoskeletal impairments are not described. This can be done with 3-dimensional instrumented gait analysis (IGA).

The purpose of IGA is to provide objective and valid measures of gait in three planes [11]. With the use of infrared camera technology and force plates embedded in the floor, it is possible to determine joint movement (kinematics), joint torque and power (kinetics) and tempo-spatial parameters. IGA thus provides a large amount of interdependent data and variables corresponding to different gait pathologies.

The quantity and complexity of data have led to the description of different indices that quantify a part of, or the overall, gait pathology into a single score. For example, the Gait Deviation Index (GDI) [12], and Gait Profile Score [13] summarise the overall gait into a single score for each patient, whereas the Gait Variable Score is an index for a single gait variable rather than a single score for all variables [13].

The use of IGA in combination with clinical examinations and standardised measures provide quantifiable information for clinical decisions regarding individually tailored interventions, in contrast to the current practice (‘care as usual’) where only clinical examinations and standardised measures are used. In the last two decades, pre-operative IGA has developed to the point where it has become an important investigation in ambulant children with CP [11, 14, 15]. Studies have shown that IGA can significantly affect the decisions regarding orthopaedic surgical interventions [16–18], and that there is good agreement between recommendations based on IGA and the surgery performed [19]. The effects of individually defined physiotherapy in children with CP based on clinical examinations and IGA have been investigated in a prospective double blind cross-over study [20]. The authors observed a superior effect of individually defined physiotherapy on achievement of treatment goals, gross motor function and some selected gait parameters compared with a generic training program. The use of IGA *per se* has only been investigated in relation to decision-making in orthopaedic surgery and effects of individually defined physical therapy.

To our best knowledge, the potential added benefit of using IGA in the decision-making of interdisciplinary interventions directed towards impairments in gait has not been investigated in children with CP. Thus, a study investigating potential difference in improvements in overall gait pathology following individually tailored interdisciplinary intervention with or without IGA is needed. The aim of this study is to determine which of two modalities (i.e. individually tailored interdisciplinary intervention with or without IGA) leads to greater improvements in the overall gait pathology, walking performance and patient-reported outcomes of functional mobility, overall health, pain and participation in normal daily activities and health-related quality of life after 52 weeks. However, it is important to note that the study is not intended to document the effect of IGA alone, but to document the difference in the effects of the interdisciplinary interventions, when IGA is implemented in the experimental group.

The primary hypothesis to be tested is:

- H^1^) The use of IGA in the planning of individually tailored interdisciplinary interventions will be more effective in improving overall gait pathology (evaluated by GDI (primary outcome)) compared with ‘care as usual’ in children with CP at GMFCS levels I and II.

The secondary hypotheses are:

- H^2^) The use of IGA in the planning of individually tailored interdisciplinary interventions will be more effective compared with ‘care as usual’ in improving walking performance (1-min walk test) and patient-reported outcomes of functional mobility (Pediatric Evaluation of Disability Inventory), overall health, pain and participation in normal daily activities (Pediatric Outcomes Data Collection Instrument) as well as health-related quality of life (Pediatric Quality of Life Inventory Cerebral Palsy Module) in children with CP at GMFCS levels I and II.

Furthermore, a number of hypothesis-generating analyses will be performed on the effects of the two modalities on the following explorative outcomes: gait, walking performance and the family-centred behaviour of health care providers.

## Methods/Design

### Study design

**Fig. 1:**
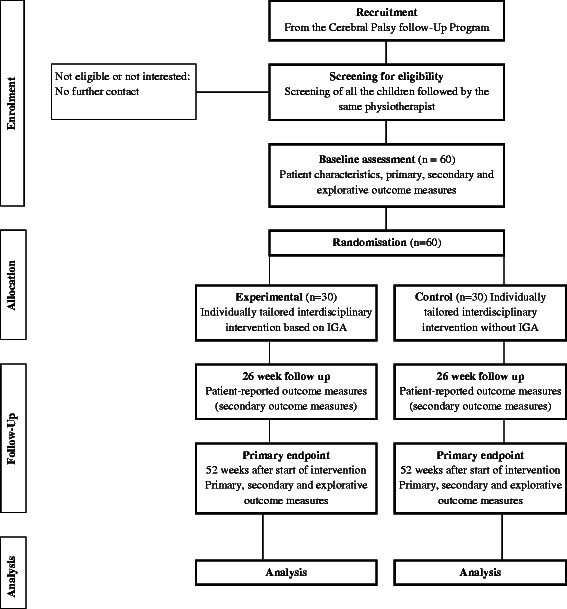
Flow diagram for the trial. The flow diagram presents an overview of the progress through the phases of the trial

A prospective, single blind, parallel group, balanced randomisation [1:1] study will be conducted in accordance with guidelines of the CONSORT statement [21, 22]. The experimental design and outcome measures are depicted in Fig. 1 and design considerations are outlined in Table 1.

**Table 1:** Design considerations. Considerations regarding the design of the study and the participants/children

Issue of consideration	Impact on study design
Compliance by patients, families and practitioners for the recommended interdisciplinary interventions	*Patients and families*Parents and the local team will receive the gait analysis report where the impairments are outlined and the recommendations are explained.
	*Practitioners*The local team will be contacted and given the opportunity to ask questions about the report and recommendations.
Risk of noncompliance with intervention amongst practitioners who are responsible for healthcare for two or more participants.	*Physical therapy* First randomised patient will undergo randomisation as described. The remaining patients followed by the same physiotherapist will be given the same allocation.
	*Orthopaedic surgery, spasticity management, orthotics* Relatively few practitioners carry out these interventions; therefore it is not possible to take into account the risk of noncompliance with the intervention amongst practitioners.
Synchronisation of interventions	Gradually, it could be assumed that methods/knowledge/attention introduced by the IGA will influence the control group. This will be evaluated post-hoc via a comparison of interventions used in the control group in the first 6 months of the study with the interventions used in the last 6 months of the study.

The current study complies with the principles of the Declaration of Helsinki. Ethics approval has been obtained from the Committee for Medical Research Ethics in the Region of Southern Denmark (S-20120162) and the Danish Data Protection Agency (2008-58-0035). Trial registration: ClinicalTrials.gov NCT02160457. Registered June 2, 2014, Update June 6, 2014.

### Participants and study setting

Participants in the Cerebral Palsy follow-Up Program in the Region of Southern Denmark and the North Denmark Region will be screened for eligibility according to inclusion and exclusion criteria described below. Written information about the study will be provided to parents and physiotherapists of eligible children by the principal investigator (HMR). Subsequently, oral information will be given to the parents of eligible children, and for those who are interested, an appointment will be scheduled for questions and further information about the study. Written consent to participate will be obtained prior to the baseline test.

Eligible participants are children aged 5 to 8 years diagnosed with spastic CP, classified at Gross Motor Function Classification System levels I or II. Exclusion criteria are: earlier interventions in the form of orthopaedic surgery within the past 52 weeks, injection with botulinum toxin type A in the 12 weeks prior to baseline assessments, and relocation to another region during the trial. Furthermore, a child will be excluded if he/she is not able to demonstrate sufficient co-operation and cognitive understanding to participate in the IGA.

This study involves six hospital units in the two regions, and the Orthopaedic Research Unit at the University of Southern Denmark. The results from the initial examination, IGA and outcome measures will be collected at the Motion Analysis Laboratory at Odense University Hospital. Patient-reported outcome questionnaires will be mailed to the parents of the participants. Interdisciplinary interventions in both groups will be conducted by the local teams at the six hospital units (paediatricians and paediatric orthopaedic surgeons) and in the 33 municipality units (physiotherapists) in the two regions. During the study period, all participants will remain in the Cerebral Palsy follow-Up Program and will receive individually tailored interdisciplinary interventions as part of the public health care system.

### Intervention

The study interventions will be carried out in two study groups:

- Experimental: Individually tailored interdisciplinary intervention based on measures performed as part of the Cerebral Palsy follow-Up Program, other clinical examinations AND IGA.
- Control: Individually tailored interdisciplinary intervention based on measures performed as part of the Cerebral Palsy follow-Up Program and other clinical examinations BUT NOT IGA (‘care as usual’).

**Fig. 2:**
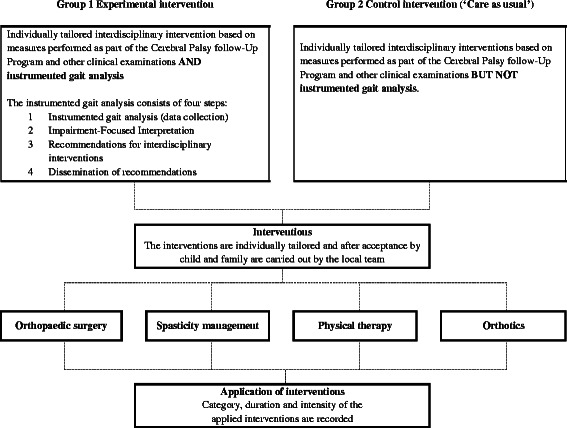
Models of individually tailored interdisciplinary interventions. This figure gives an overview of the two models of individually tailored interdisciplinary interventions that are under investigation in the study

The two models of individually tailored interdisciplinary intervention are outlined in Fig. 2. The trial is not designed to distinguish between the different elements in the two intervention groups.

For both the experimental and control groups, the interdisciplinary interventions addressing impairments that affect the patients’ gait, can be described in four categories [5, 6]:

- *Orthopaedic surgery*, such as tendon transfer, muscle tendon lengthening, rotational osteotomy and stabilisation of joints that aim to restore joint mobility, muscle function, stability and lever arm dysfunction [23].
- *Spasticity management*, where the most frequently used intervention is injection of botulinum toxin type A in muscles with increased muscle tone in the lower extremities [24].
- *Physical therapy* such as goal-directed training or functional training of specific elements of the gait or walking [6].
- *Orthotics*, such as ankle-foot orthoses that provide stability and/or mobility of the joints and/or support muscle function [25].

The study will not involve standardisation of the interdisciplinary intervention and will not provide training in the interventions provided by the participating hospitals and municipalities. This is to ensure a pragmatic approach to reflect common practice and ensure high external validity of the study.

#### Experimental

The experimental intervention will include an individually tailored interdisciplinary intervention based on clinical examinations, standardised measurements of walking and recommendations for interventions based on knowledge about the impairments that affects the gait from IGA. An interdisciplinary team will provide recommendations for interventions based on impairment-focused interpretation and reporting according to Baker 2013 [26]. The data collection, interpretation, development of recommendations and dissemination of recommendations will be carried out in four steps:

##### Step 1: Instrumented gait analyses (data collection)

Instrumented gait analysis including clinical examination, sagittal and coronal plane video recording and 3-dimensional kinematics and kinetics will be carried out. An 8-camera Vicon T40 system (Vicon, Oxford, UK) operating at 100Hz will be used for data collection. Ground reaction forces will be recorded using two force plates (AMTI, OR6-7-1000, Watertown, MA, USA), sampling at 1000Hz. The Plug-in Gait model, Vicon Nexus Software (version 1.7.1 or later) and Vicon Polygon software (version 3.5.2 or later) will be used for data processing, to define gait cycles, spatio-temporal parameters, kinematic and kinetic data [27]. The children will walk barefoot and, if relevant, also with orthotics and shoes, at a self-selected speed along a 10-m walkway until at least five acceptable trials are collected for each child. To validate the gait performance, parents will be asked if the gait is representative of their child’s normal walking.

##### Step 2 Impairment-focused interpretation

The approach ‘Impairment-Focused Interpretation’ [26] refers to the interpretation of the gait analysis. The principal investigator (HMR) will identify and describe the impairments that are affecting the child’s gait in a standardised report and subsequently validate findings with the head of the motion laboratory (AHL).

##### Step 3: Recommendations for interdisciplinary interventions addressing impairments from IGA

The recommendations will address the impairments found in the impairment-focused interpretation (*Step 2*) and will be provided by the gait analysis team, which will consist of a neuro-paediatrician (LKH), a paediatric orthopaedic surgeon (NWP or VE), a physiotherapist (HMR) and a biomechanist (AHL). To facilitate an objective recommendation for treatment selection based on treatment algorithms described by Miller 2007 [28], we created a list of the most common underlying neuro-musculoskeletal impairments of the primary movement features found in IGA (see Table 2). Finally, each of the recommendations for interdisciplinary interventions will be based upon consensus. Otherwise, the specific interventions will not be recommended.

**Table 2:** Considerations before recommending interdisciplinary interventions. To facilitate an objective recommendation for treatment selection, we created a list of the most common underlying neuro-musculoskeletal impairments of the primary movement features found in IGA. The table describes the primary segment of movement feature (column 1), underlying neuro-musculoskeletal impairment (column 2–3) and the interdisciplinary interventions under consideration (column 4–7)

Primary segment of movement feature and underlying neuro-musculoskeletal impairment			Interdisciplinary interventions under consideration			
			Orthopaedic surgery	Spasticity management	Physical therapy	Orthotics
**Pelvic**						
	*Altered range of movement in anterior/posterior tilt, caused by impairments in:*					
		Body structures	x			
		Muscle tone function		x		
		Muscle power or endurance function			x	
	*Altered range of movement in pelvic obliquity, caused by impairments in:*					
		Body structures (Limb length discrepancies)	x			x
	*Compensation for reduced control of movements*				x	
**Hip**						
	*Altered range of movement in flexion/extension, caused by impairments in:*					
		Body structures	x		x	
		Muscle tone function		x		
		Muscle power or endurance function			x	x
	*Altered range of movement in abduction/adduction, caused by impairments in:*					
		Body structures	x		x	
		Muscle tone function		x		
		Muscle power or endurance function			x	x
	*Altered range of movement in rotation, caused by impairments in:*					
		Body structures	x			
		Muscle tone function		x		
		Muscle power or endurance function			x	x
	*Compensation for reduced control of movements*				x	
**Knee**						
	*Altered range of movement in flexion/extension, caused by impairments in:*					
		Body structures	x		x	
		Muscle tone function		x		
		Muscle power or endurance function			x	x
	*Compensation for reduced control of movements*				x	x
**Ankle and foot progression**						
	*Altered range of movement in dorsi- or plantarflexion, caused by impairments in:*					
		Body structures	x		x	x
		Muscle tone function		x		
		Muscle power or endurance function			x	x
	*Altered range of movement in foot progression, caused by impairments in:*					
		Body structures and/or function (Tibial torsion, Planovalgus, Equinovarus))	x			x
	*Compensation for reduced control of movements*				x	x

##### Step 4: Dissemination of recommendations to the child, family and local team

The parents of the child and the local team, which consists of a paediatrician, a paediatric orthopaedic surgeon, a physiotherapists and/or an orthotist, will be informed about the recommendations for interventions based on knowledge from IGA. To promote the application of the recommended intervention from IGA, members of the local team will be asked if they have any questions about the results of the report and whether they will follow the recommendations. Furthermore, they will be asked which specific goals they have set for the applied interventions.

Adherence to the recommended interventions is not a prerequisite for participation in the study. As in daily clinical practice, the child, his/her family and the local team will have the option to follow or to reject the recommended intervention or to choose other interventions than those recommended by the gait analysis team.

#### Control

The control intervention (‘care as usual’) will include individually tailored interdisciplinary interventions based on measures performed as part of the Cerebral Palsy follow-Up Program and other clinical examinations, but not the IGA.

### Measurements

All patient characteristics and outcomes are listed in Table 3. Patient characteristics, IGA and 1-min walk will be performed at baseline and at 52 weeks post start of intervention (primary endpoint). The patient-reported outcome measures will be conducted at baseline, 26 weeks, and 52 weeks post start of intervention. The time point ‘start of intervention’ is defined as the week where the report is released. The data collection in the control group will be adjusted according to the planned time points in the experimental group. Furthermore, to acknowledge that surgery might be influenced by a long planning phase (i.e. consideration of surgery, involvement of patient and family and planning) and rehabilitation, a second post intervention examination will be performed at 52 weeks post operation and included in a per protocol analysis.

**Table 3:** Summary of measures to be collected. All patient characteristics and outcomes to be collected at baseline, 26 weeks and at primary endpoint (52 weeks) are listed in the table

		Instrument	Baseline	26 weeks	Primary endpoint
**Baseline data and classification of function**					
	Age (years)		x		
	Ability to carry out self-initiated movements	GMFCS	x		x
	Functional mobility	FMS	x		x
	Height (cm)		x		x
	Leg length (cm)		x		x
**Primary outcome measure: Gait**					
	Overall gait pathology	GDI	x		x
**Secondary outcome measures**					
	Walking performance (metre)	1-min walk	x		x
	Functional mobility	PEDI	x	x	x
	Health-related quality of life	PedsQL	x	x	x
	Overall health, pain and participation	PODCI	x	x	x
**Explorative outcome measures** Walking performance, gait pathology, spatio-temporal parameters, and behaviour of health care providers					
	Gait pathology	GVS	x		x
	Step length and time Stride length and cadence Time of single support for each leg and double support Walking speed	IGA	x		x
	Family-centred behaviour of health care providers	MPOC-20	x	x	x
**Recommended and applied interventions**					
	Categories of recommended interventions		x		
	Categories of applied interventions			x	x

*Abbreviations:* *GMFCS* Gross Motor Function Classification System, *FMS* Functional Mobility Scale, *IGA* Instrumented gait analysis, *GDI* Gait Deviation Index, *1-min walk* 1 min Walk Test, *PEDI* Pediatric Evaluation of Disability Inventory, *PedsQL* Pediatric Quality of Life Inventory Cerebral Palsy Module^TM^, *PODCI* Pediatric Outcome Data Collection Instrument, *GVS* Gait Variable Score, *MPOC-20* Measure of Processes of Care, *CPUP* Cerebral Palsy follow-Up Program

In addition to the baseline data and classification, primary and secondary outcome measures, a range of exploratory outcome measures will be collected. The primary and the secondary outcome measures will be used to confirm or reject the described hypotheses, while the explorative outcome measures will be used for hypothesis generation, and to report other potential beneficial or harmful effects of the interventions.

### Baseline data and classification of function

The Gross Motor Function Classification System (GMFCS) will be used to classify the child’s ability to carry out self-initiated movements related to sitting and walking [9]. The GMFCS has strong construct validity with the Gross Motor Function Measure (GMFM) [29] and good inter-observer and test-retest reliability with generalisability coefficient values of 0.93 and 0.79 [30]. Furthermore, the Functional Mobility Scale will be used to quantify the child’s mobility according to the need for assistive devices in different environmental settings [10]. Construct validity has been investigated and inter-observer reliability with agreement values of 0.86 to 0.92 with weighted kappa coefficients have been shown [31, 32].

#### Primary outcome measure

##### Overall gait pathology

IGA will be conducted as described in *Step 1: Instrumented gait analyses.* Data from five representative trials will be analysed. Both at baseline and post intervention, the data collection will be done at a self-selected walking speed. If the self-selected walking speed on the two occasions differs more than 15 %, the data collection will also be conducted at a walking speed matched to that at baseline. A trained lab technician will perform the data collection and data processing.

The primary outcome measure is the GDI, which is based upon kinematic data from the IGA, and is an overall quantitative index that summarises the overall gait pathology into a single score for each patient by comparison with non-pathological gait. A GDI value of 100 represents the absence of gait pathology, and each 10-point decrement below 100 indicates one standard deviation from normal gait kinematics [12]. For the primary outcome measure, the median of the five trials for each leg will be used to calculate the average of both legs to provide a single index for each child. Since gait speed *per se* might affect GDI, the primary outcome analysis will be based upon matched walking speed, as described above.

Satisfactory concurrent and construct validity of the GDI in children with CP have been shown [12, 33]. The GDI has demonstrated excellent intra-rater reliability and acceptable agreement across two repeated sessions in children with CP [34]. The responsiveness of GDI has been shown by comparing the GDI score before and after surgical lengthening of the gastrocnemius in children with CP [35].

#### Secondary outcome measures

##### Walking performance

Walking performance will be measured by using the 1-min walk test and will be performed as described by McDowell et al. [36]. It has demonstrated high correlation with gross motor function [37] and good test-retest reliability with ICC values of 0.94 for children with CP [36].

##### Functional mobility

The Mobility Scale of the original Pediatric Evaluation of Disability Inventory evaluates the child’s functional mobility in everyday activities with regard to functional skills and amount of caregiver assistance [38]. A Danish version will be applied as a parental questionnaire: The content and discriminative validity have been established in children with CP [39, 40].

##### Health-related quality of life

The Pediatric Quality of Life Inventory Cerebral Palsy Module is a measure of health-related quality of life, specifically designed for children with CP. It is based upon the parents’ report and measures physical, emotional, social and school functioning. Construct and discriminative validity of the original version have been supported by comparing the scores from children with CP with a generic measure of the same construct with those from children without disability. Satisfactory reliability with ICC values of 0.42 to 0.84 was demonstrated in the same study [41]. A linguistically validated Danish version will be used [42].

##### Overall health, pain and participation

The Pediatric Outcomes Data Collection Instrument assesses overall health, pain and participation in normal daily activities. Concurrent and discriminant validity have been assessed by comparing the Pediatric Outcomes Data Collection Instrument with other measures of health and well-being, gross motor function and diagnostic subgroups in children with CP [43]. Moderate to good test-retest reliability with ICC values of 0.71 to 0.97 has been reported in children with orthopaedic or musculoskeletal disorders [44]. The Pediatric Outcomes Data Collection Instrument is currently being translated into Danish.

#### Exploratory outcome measures

##### Gait pathology

Data from the IGA will be used to calculate the median Gait Variable Score of the first five trials for each leg at a self-selected walking speed and at matched (pre and post) walking speed, to identify changes in gait pathology at joint levels. The explorative outcome measures based upon the Gait Variable Score will be used for hypothesis-generation purposes.

Satisfactory face and criterion validity of the Gait Variable Score in children with CP have been shown [45]. Investigation of intra-session variability has suggested that the Gait Variable Score is a reliable measure within a single session [13]. Fair to good intra-rater reliability and acceptable agreement across two repeated sessions have been shown for the Gait Variable Score in children with CP [34].

##### Walking performance

The following spatio-temporal parameters from the IGA will be used:

1. Step length and time, and limb-to-limb asymmetry index,
2. Stride length and cadence,
3. Time of single support for each leg and double support, and limb-to-limb asymmetry index, and
4. Walking speed.

Intra-subject reliability of gait analysis in normal and spastic children has been investigated. The study reported acceptable coefficients of variation of 3.4 to 9.7 % on spatio-temporal parameters in children with spastic CP [46].

##### Family-centred behaviour of health care providers

Measure of Processes of Care is a self-report measure of parents’ perception of the extent to which the health services that their child receives are family-centred. Concurrent validity has been investigated by comparison with measures of satisfaction and stress. Discriminative validity has been demonstrated by comparing Measure of Processes of Care scores between different programs of service delivery and acceptable reliability with Cronbach’s alpha of 0.83 to 0.90 has been documented [47]. A Danish version will be used [48].

##### Recommended and applied interventions

Records of the recommended and applied interventions will be used to explore the type and number of interventions in the two groups with regard to category (Orthopaedic surgery, Spasticity management, Physical Therapy and Orthotics).

#### Adverse events

Any adverse events that occur in the experimental and control groups will be registered and reported in accordance with the standards of the Danish Health and Medicines Authority. Information about adverse events will be gathered from parents of the participants, from the local teams and from the gait laboratory staff. Adverse events may occur as a direct result of the study activities, such as a fall during the IGA or indirectly as a result of the interdisciplinary interventions, such as pressure sores after casting. Any detected adverse advents or unintended effects will be reviewed by the principal study investigator (HMR) and by a neuro-paediatrician (LKH). The events will be listed and defined, with reference to standardised criteria where appropriate.

### Sample size

The sample size for this study is calculated to create power for the primary hypothesis. The sample size calculation is based upon the GDI (primary outcome), collected as part of another study in our laboratory on a comparable group of children with CP (mean GDI 79.3, SD 12.0). A minimum clinically important difference in GDI has been defined as 7.9 points by the current group of authors a priori, which is equivalent to an improvement of 10 %, as suggested by Swartz et al. [49] . A minimum of 29 subjects in each group (*n* = 58) is required with alpha = 0.05 and 80 % power. Following these estimations, it was decided to include 60 children in total (30 patients in each group), allowing for a dropout rate of 5 %.

### Randomisation

After baseline assessment, children will be randomised to either the ‘Experimental’ or the ‘Control’ group. The randomisation will be stratified according to the physiotherapist to whom the child is appointed. For children who are followed by a physiotherapist, who is responsible for two or more children, the first child randomised will determine how the following children will be allocated.

Randomisation will be computer-generated by a researcher with no other involvement in the study. Participants will be allocated by a sequence of numbers: 0 – referring to ‘Experimental’, and 1 – referring ‘Control’. The allocation sequence will be concealed in sequentially numbered opaque, sealed envelopes. When all participants followed by the same physiotherapist have completed the baseline assessment the principal investigator (HMR) will open the envelope and inform the child’s parents and the local team about the allocation.

### Blinding

Participants and the local team will not be blinded. Data collectors and data analysts will be blinded.

### Data and statistical analysis

Main comparative analyses between groups will be performed using an intention-to-treat analysis (all cases with available baseline data carried forward). Between-group mean differences and 95 % confidence intervals will be estimated with a linear model in which baseline scores are entered as the only covariate [50, 51]. Model specifications will depend on evaluation of distributional properties of collected data and appropriate adaptation of point estimate and variation indicators. Data analysis will be performed on the groups of children randomised first and for the whole group of children to explore any differences with regard to whether a child was randomly assigned to the intervention or followed another child in the randomisation.

Secondly, a per protocol analysis will be performed. Proportional odds models will compare the difference between the two groups based on the participant-perceived response to treatment.

## Discussion

To our knowledge, this is the first randomised controlled trial investigating the effectiveness of an individually tailored interdisciplinary intervention addressing impairments identified by IGA compared with ‘care as usual’ in children diagnosed with CP. Such a trial is warranted because IGA is widely used for orthopaedic surgical planning [11, 14, 15] and has been shown to affect the decision-making in the planning of orthopaedic surgery [18]. However, its effectiveness regarding gait pathology, walking performance and patient-reported outcomes of functional mobility, overall health, pain and participation in normal daily activities as well as health-related quality of life have never been investigated.

The IGA has been investigated for quality as a measurement tool [12, 33, 35, 46, 52]. The current trial seeks to investigate the effectiveness of the IGA when applied in a clinical practice involving multiple steps such as interdisciplinary interventions in regard to changes in the overall gait pathology, walking performance and patient-reported outcomes of functional mobility, overall health, pain and participation in normal daily activities and health-related quality of life after 52 weeks. Consequently, the current trial uses a pragmatic approach and is accordingly not designed to distinguish between the different elements in the two intervention groups but rather to reflect common practice and ensure high external validity. This is in contrast to studies emphasising internal validity that are carried out in an ‘ideal setting’ with highly selected participants, practitioners and hospitals [21].

The randomised controlled trial design will be used to assess potential benefits associated with the use of the IGA in interdisciplinary interventions, and thereby, provide novel evidence. The randomised controlled trial design is considered the gold standard for a clinical trial, and provides the most reliable evidence on the efficacy of healthcare interventions [22]. The study can be used to support the decision-making as to whether IGA should be applied in routine daily practice to all children with spastic CP at GMFCS levels I and II. Thus, the purpose of the study warrants a pragmatic approach as opposed to a more explanatory design. The key differences in the two approaches can be described in terms of purpose, setting, participants, intervention and outcomes [21], which will be incorporated in the following sections of the discussion.

The study will be carried out in the Region of Southern Denmark and the North Denmark Region. Participants will be recruited through the local teams in the Cerebral Palsy follow-Up Program, and will encourage attendance among eligible children. The Cerebral Palsy follow-Up Program makes it possible to gain information to make a thorough description of the ‘reach’ of recruitment of participants into the population of interest and to document potential study composition differences across the stages of the trial [22]. The relatively young age group has been chosen to ensure inclusion of children at an early age, before the development of extensive and fixed deformities that cause impairments and associated gait pathology [53]. To ensure good data quality from IGA, participants at GMFCS levels I and II have been chosen. However, this may impact the generalisability of findings.

To reflect the current clinical procedures in Denmark and to emphasise external validity, the experimental intervention will be carried out in five steps. Selected practitioners, who are highly trained, are responsible for the first three steps (*Step 1: IGA, Step 2: Impairment-focused interpretation, and Step 3: Recommendations for interdisciplinary interventions*). Both the selected practitioners and the local team will be involved in the remaining step (*Step 4: Dissemination of recommendations*) and planning of individually tailored interdisciplinary interventions. Paediatric orthopaedic surgeons will perform the orthopaedic surgical procedures while the local teams will carry out other interventions in terms of spasticity management, physical therapy and orthotics. Consequently, only parts of the experimental intervention (*Steps 1,2 and 3*) will be standardised and strictly enforced by researchers responsible for the study, whereas the remaining parts of the experimental interventions will be performed through the collaboration of local teams, the selected practitioners and the researchers. The local teams, regardless of treatment group, will use their standard procedures in the interdisciplinary interventions.

There is a risk of poor adherence to the recommended interventions by participants and local teams. This has previously been reported in a randomised controlled trial that investigated the impact of gait analysis on surgical outcomes in ambulatory children with CP, where less than half (42 %) of the IGA recommendations were followed [54]. To improve understanding of the recommended interventions from the IGA, members of the local team will be asked if they have any questions about the results of the report and whether they will follow the recommendations. The identification of individually tailored treatment goals has previously been used in studies concerning physical therapy [55, 56] and orthopaedic surgery [57] for children with CP. Studies have shown that the approach can promote improvement in everyday activities and gross motor function [55], and that the approach resulted in goals that were more frequently and smoothly implemented [56].

As for the majority of studies that involve interventions that cannot be blinded, the current study design has a potential risk of non-compliance of participants with the intervention they are randomised to. In this study, there is a risk that participants randomised to the control intervention (‘care as usual’) will benefit from knowledge obtained by practitioners from participants in the experimental intervention. However, since IGA is only performed in our institution, no one will gain access to the examination without our knowledge. Thus, the risk of non-compliance is primarily believed to be at the physiotherapist level. Consequently, as described above, in cases where physiotherapists are responsible for the interventions for two or more participants, the first randomised patient will determine the allocation of the following patients. The interventions performed at the level of orthopaedic surgery, spasticity management and orthotics will be carried out by relatively few practitioners. Thus, it will be easier to contain this risk and practitioners will simply be requested to continue with their standard care for the ‘care as usual’ group. These professionals have taken part in the IGA interpretations for a number of years. A consequence of this set-up might be that the interventions in the ‘care as usual’ group could be influenced by the professional experience gained from previous interpretations of the IGA.

We have chosen a follow-up period of 52 weeks. This is done to balance the desire for a short follow up for the interventions’ spasticity management and physical therapy, while the effects of orthopaedic surgery and orthotics might take as long as 24 months to emerge [58].

A wide range of outcome measures has been used to document the effectiveness of interventions in children with CP [43, 44]. For this study, we have decided to include assessments of body function and structure, activity and participation levels from the International Classification of Functioning, Disability and Health [59]. The primary outcome measure is at the level of ‘body function’, where we use overall gait pathology classified by the GDI as a measure of gait pattern functions that are defined as functions of movement patterns associated with walking [59] and can be used to reflect the extent to which the goal of ‘better looking gait’ has been reached [60]. Performance on the GDI was chosen as the primary outcome rather than on the Gait Profile Score, because it seems to be more sensitive to change in children with a relatively mildly affected gait [13], as expected with the study population of children at GMFCS levels I and II. The gait pattern function has been found to be one of the important domains for youth with CP, parents and medical professionals, when considering treatment outcomes [60].

The secondary outcome measures are a range of measures on the level of ‘activity and participation’. The measures have been chosen to be relevant to the particular group of children participating in the study. The Gait Variable Score will be calculated to document changes in the nine kinematic variables and will be used to document explorative changes at the joint level. Thus, we have chosen a wide range of outcome measures that covers all levels of the International Classification of Functioning, Disability and Health and seems relevant to participants, their parents and the healthcare professionals.

One might argue that the Gross Motor Function Measure [8] could be a relevant outcome measure. However, due to the time-consuming IGA procedure, it would be difficult to motivate the children for further examination and, consequently, difficult to achieve valid output measures. Furthermore, there is a risk of a ceiling effect when the Gross Motor Function Measure is used for children at GMFCS level I after the age of five years, due to their relatively high level of functioning.

The current trial will provide novel evidence for the effects of an individually tailored interdisciplinary intervention designed to address impairments identified by IGA versus ‘care as usual’ in children with spastic CP. The results of the trial will be submitted to peer-reviewed journals for publication, irrespective of the outcome, in accordance with the CONSORT statement for the reporting of randomised controlled trials.

## Abbreviations

CP: cerebral palsy
GDI: gait deviation index
GMFCS: gross motor function classification system
ICC: intra-class correlations coefficient
IGA: instrumented gait analysis
